# The Implications of PDK1–4 on Tumor Energy Metabolism, Aggressiveness and Therapy Resistance

**DOI:** 10.3389/fonc.2020.583217

**Published:** 2020-12-15

**Authors:** Emine Atas, Monika Oberhuber, Lukas Kenner

**Affiliations:** ^1^ Department of Pathology, Medical University of Vienna, Vienna, Austria; ^2^ Area ‘Data & Technologies’, CBmed—Center for Biomarker Research in Medicine GmbH, Graz, Austria; ^3^ Unit of Pathology of Laboratory Animals, University of Veterinary Medicine Vienna, Vienna, Austria; ^4^ Christian Doppler Laboratory for Applied Metabolomics (CDL AM), Division of Nuclear Medicine, Department of Biomedical Imaging and Image-Guided Therapy, Medical University of Vienna, Vienna, Austria

**Keywords:** pyruvate dehydrogenase kinase, tricarboxylic acid cycle, oxidative phosphorylation, Warburg effect, aerobic glycolysis, prostate cancer, cancer metabolism, therapy resistance

## Abstract

A metabolic shift from oxidative phosphorylation (OXPHOS) to glycolysis—known as the Warburg effect—is characteristic for many cancers. It gives the cancer cells a survival advantage in the hypoxic tumor microenvironment and protects them from cytotoxic effects of oxidative damage and apoptosis. The main regulators of this metabolic shift are the pyruvate dehydrogenase complex and pyruvate dehydrogenase kinase (PDK) isoforms 1–4. PDK is known to be overexpressed in several cancers and is associated with bad prognosis and therapy resistance. Whereas the expression of PDK1–3 is tissue specific, PDK4 expression is dependent on the energetic state of the whole organism. In contrast to other PDK isoforms, not only oncogenic, but also tumor suppressive functions of PDK4 have been reported. In tumors that profit from high OXPHOS and high *de novo* fatty acid synthesis, PDK4 can have a protective effect. This is the case for prostate cancer, the most common cancer in men, and makes PDK4 an interesting therapeutic target. While most work is focused on PDK in tumors characterized by high glycolytic activity, little research is devoted to those cases where PDK4 acts protective and is therefore highly needed.

## Introduction

**Figure 1:**
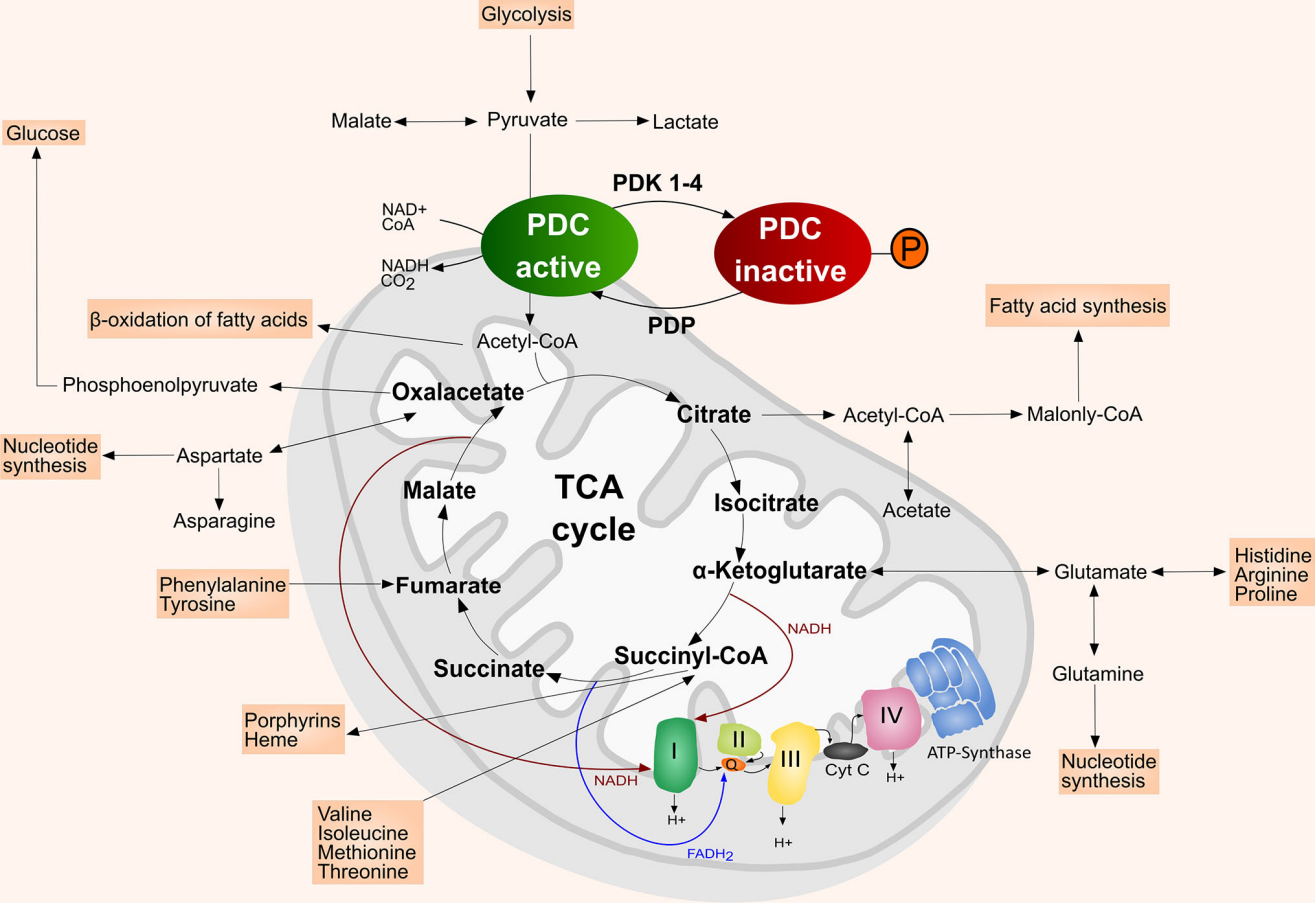
Simplified scheme of the mitochondrion with TCA cycle and the intersecting anaplerotic and cataplerotic reactions, OXPHOS complexes I–IV, and ATP synthase (complex V). In the mitochondrial matrix the PDC catalyzes the irreversible conversion of pyruvate, NAD+ and CoA into acetyl-CoA, NADH and CO_2_. PDK inactivate the PDC by phosphorylating its E1*α* subunit, which hinders the entrance of acetyl-CoA into the TCA cycle. The PDC is reactivated upon dephosphorylation by PDP. Adapted from (5). Inspiration (4, 6).

Besides other hallmarks of cancer, such as sustained proliferative signaling, resistance to cell death, invasiveness and increased angiogenesis, tumors are characterized by their altered metabolic features (1, 2). The two major metabolic pathways providing energy in the form of adenosine triphosphate (ATP) are glycolysis and oxidative phosphorylation (OXPHOS) (1, 3). Under aerobic conditions, glucose is metabolized to pyruvate *via* glycolysis in the cytosol (4). Pyruvate is then processed to CO_2_ in the mitochondria *via* the tricarboxylic acid (TCA) cycle and OXPHOS (1, 3) (**Figure 1**). On the contrary, under anaerobic conditions glycolysis is favored, where pyruvate is mostly converted to lactate and only minimal amounts enter the TCA cycle (1, 3). Cancer cells typically present a metabolic shift from the TCA cycle/OXPHOS to glycolysis or lactate fermentation regardless of the presence of oxygen, a phenomenon known as the “Warburg effect” (7, 8). Hereby, cancer cells obtain survival advantages in hypoxic tumor microenvironments where OXPHOS is compromised (3). However, the switch to aerobic glycolysis in the cancer cell is not only limited to hypoxia but is also activated by deregulated signals enhancing glycolysis or hindering OXPHOS (3, 9, 10).

The metabolic shift from TCA cycle/OXPHOS to aerobic glycolysis is tightly regulated (11). The following review focuses on the key players in this regulation—the mitochondrial pyruvate dehydrogenase complex (PDC) and the pyruvate dehydrogenase kinases (PDK). PDK have been associated with tumor aggressiveness, proliferation, anti-apoptotic effects and therapy resistance in numerous malignancies (12–16). We here provide a compact overview on the latest research on the cancer specific levels of PDK isoforms and their associations with tumor aggressiveness and therapy resistance. We will also address the remarkable energy metabolism of prostate cancer (PCa) and the resulting effects of PDK on tumor growth. A detailed discussion of metabolic pathways intersecting with the TCA cycle and their interplay with the PDC/PDK axis is beyond the scope of this review but can be found here: Gray et al., Hirschey et al., Martínez-Reyes and Chandel et al., Vander Heiden and DeBerardinis et al. (4, 17–19).

## Regulation of the Metabolic Shift by PDC/PDK Activity

One of the main enzymes regulating the metabolic shift in mammals is the mitochondrial PDC (20, 21). It is composed of the pyruvate dehydrogenase (E1), dihydrolipoamide acetyl-transferase (E2), dihydrolipoamide dehydrogenase (E3) and the E3 binding protein (E3BP) (4, 22). The PDC catalyzes the irreversible conversion of pyruvate, nicotinamide adenine dinucleotide (NAD+) and coenzyme-A (CoA) into acetyl-CoA, NADH and CO_2_ (4). The converted acetyl-CoA then enters the TCA cycle (4). Thus, PDC represents an important link between glycolysis and TCA cycle/OXPHOS (20, 21, 23) (**Figure 1**). PDC is more active in the healthy and well-nourished state, whereas its activity is decreased during fasting or low glucose levels, but also in diabetes and most cancer types (21, 24). The activity of the PDC is mainly regulated by four PDK isoenzymes (PDK1–4) that are located in the mitochondrial matrix (20, 25). PDK1–4 achieve a reversible downregulation of the PDC by phosphorylating specific serine residues (Ser293, Ser300, and Ser232) of its E1*α* subunit, thereby reducing the metabolic flux through the PDC and downstream pathways (4, 20, 23, 25). The E1*α* subunit of the PDC can be dephosphorylated by pyruvate dehydrogenase phosphatase (PDP), which leads to the reactivation of the PDC (4, 22, 23) (**Figure 1**). In addition, PDC can also be reversely acetylated and succinylated (26, 27). Acetylation of the PDC E1*α* subunit by acetyl-CoA acetyltransferase 1 (ACAT1) results in dissociation of PDP1 from the PDC and PDK1 recruitment, thereby suppressing PDC activity (26, 28, 29). Lysine desuccinylation of PDC subunits by sirtuin (SIRT) 3 and SIRT5 also results in suppression of PDC activity (26).

Besides generating reductive equivalents for OXPHOS, the TCA cycle provides precursors for biosynthetic processing of lipids, amino acids, and nucleotides (17, 19). Anaplerotic (carbon replenishing) and cataplerotic (carbon expending) pathways intersecting the TCA cycle balance the carbon flux (4). Pyruvate provides carbon either *via* the PDC or alternatively *via* conversion to oxaloacetate (4, 17, 30). Furthermore, glutamine contributes glutamate, α-ketoglutarate, aspartate, CO_2_, pyruvate, lactate, alanine and citrate to the TCA cycle, which makes it a key player in the mitochondrial metabolism supporting proliferation of cancer cells (**Figure 1**) (4, 17, 19, 30). A detailed discussion of the role of glutamine metabolism in cancer was published by Masisi et al. (31).

## PDK1–4 Levels in Different Cancers and Their Prognostic Implications

PDK1–4 are differentially expressed in several metabolic tissues (32). PDK1 is abundant in the cardiac muscle, pancreatic islets, and skeletal muscle and is expressed at lower levels in other tissues (20, 22, 23, 33–35). PDK2 on the contrary is ubiquitously expressed, with the highest expression levels in the heart, diaphragm, kidney, and red skeletal muscles (22). Other tissues such as liver, brain, testis, ovaries, and lung show lower PDK2 protein levels (22). While PDK3 has a weak expression pattern in kidney, brain, testis, and lung, PDK4 is mainly expressed in the heart, skeletal muscle, pancreatic islets and at intermediate levels in the liver, lung, and kidney (20, 22, 23, 33–35).

PDK1, a downstream target of hypoxia inducible factor 1 alpha (HIF1*α*), is upregulated in a number of cancers including ovarian cancer (OCa) (36), gastric cancer (GCa) (37, 38), colorectal cancer (CRCa) (39), PCa (40), and acute myeloid leukemia (AML) (41). Involvement of PDK1 has also been implicated in cancer cell epithelial–mesenchymal transition (EMT) and metastasis, for example in metastasis of liver aggressive 4T1 breast cancer (BrCa) cells to the liver, which implies an oncogenic role (12) (**Table 1**). PDK1 can be tyrosine phosphorylated and thereby activated by tyrosine kinase fibroblast growth factor receptor 1 (FGFR1), which localizes to mitochondria (64). Interestingly, both the PDC and PDK1 were also detected in the outer mitochondrial matrix, where PDK1 can be directly phosphorylated by tyrosine kinases (64).

**Table 1:** Overview of PDK1–4 expression levels in different cancer types, their effect on prognosis upon up-regulation and tumorigenesis.

Expression level in cancer				Prognosis upon up-regulation	Effect on tumorigenesis	References
**PDK1**	AML	↑		p. bad	oncogenic	(41)
	BrCa	↑		bad	oncogenic	(12, 42)
	CRCa	↑		p. bad	oncogenic	(39)
	GCa	↑		bad	p. oncogenic	(37, 38)
	HNSCCa	↑		bad	oncogenic	(43, 44)
	NSCLCa	↑		bad	oncogenic	(45)
	OCa	↑		bad	oncogenic	(36)
	PCa	↑		p. bad	oncogenic	(40)
**PDK2**	AML	↑		bad	p. oncogenic	(46)
	CRCa	↑		bad	p. oncogenic	(14)
	GCa	↑		p. bad	oncogenic	(47)
	HCCa	↑		p. bad	oncogenic	(48)
	HNSCCa	↑		p. bad	oncogenic	(49, 50)
**PDK3**	AML	↑		bad	p. oncogenic	(46)
	ChCa	↑		bad	p. oncogenic	(51)
	CRCa	↑		bad	oncogenic	(15)
	GCa	↑		p. bad	oncogenic	(52)
	Glioma	↑		p. bad	oncogenic	(53)
	Melanoma	↑		p. bad	oncogenic	(54)
	PCa	↑		p. bad	oncogenic	(40)
**PDK4**	AML	↑		p. bad	oncogenic	(55)
	BlCa	↑		p. bad	oncogenic	(56)
	BrCa	↑		bad	p. oncogenic	(57, 58)
	CRCa	↑		bad	oncogenic	(13, 59)
	HCCa		↓	good	suppressive	(16, 60)
	NSCLCa		↓	p. good	suppressive	(61)
	OCa	↑		bad	oncogenic	(62)
	PCa		↓	good	p. suppressive	(63)

p. = presumably, ↑ = high, ↓ = low.

PDK2 is the only PDK enzyme that has been confirmed as p53 target (65). P53 downregulates and controls PDK2 expression on transcriptional and posttranscriptional level and thereby reduces the Warburg effect (25, 65). In hepatocellular carcinoma (HCCa) (48) and GCa cells (47) proliferation and migration were suppressed after downregulation of PDK2. PDK2 has also been associated with therapy resistance in CRCa cells (14), in head and neck squamous cell carcinoma (HNSCCa) cells (49), and in non-small cell lung cancer (NSCLCa) patients (66) (**Table 1**).

PDK3, which has the highest binding affinity to the PDC, is the least studied isoenzyme of the PDK (25). Similarly to PDK1, PDK3 is induced by HIF-1*α*, and higher expression is associated with higher tumor stage in many cancers (15, 67). In GCa (52), glioma (53), PCa (40), AML (46), and melanoma (54), high expression of PDK3 has been shown. Knockdown of PDK3 in the GCa cell lines SGC7901 and AGS (52), and the PCa cell line LNCaP (40) inhibited proliferation and induced apoptosis. Moreover, elevated expression of PDK3 is associated with chemo resistance in GCa cells (68), increased drug resistance in CoCa cells (15) and correlates with poor prognosis in cholangiocarcinoma (ChCa) (51), and AML (46) (**Table 1**).

While the regulation of PDK1–3 reflects the immediate energy demands of the cell, PDK4 reflects whole organism energy balance and is upregulated during excessive exercise (69), starvation (70), in insulin resistant states and diabetes (6, 57). PDK4 is also involved in the control of muscle size in cancer stages or after chemotherapy treatment, which renders it interesting as a target to combat cancer-associated cachexia (20). Based on the metabolic function of the respective tissue, the cancer type and stage, high PDK4 expression can act either oncogenic or tumor suppressive, as described below (**Table 1**).

The overexpression of PDK4 is associated with poorer prognosis in BrCa patients, irrespective of their molecular or histological subtype (58) and is associated with antiestrogen resistance (57). Duan et al. showed that PDK4 expression induced by benzyl butyl phthalate promotes glycolysis and proliferation in AML cells (55). In human metastatic CoCa cells, knockdown of PDK4 reduced their migratory and invasive properties (13). Furthermore, HIF1*α* expression was reduced in PDK4 knockdown cells, suggesting a correlation between PDK4 and HIF1*α* (13). PDK4 is also linked to enhanced cell proliferation and invasion in OCa (62) and bladder cancer (BlCa) (56). In addition, PDK4 has been identified as a positive regulator and activator of mechanistic target of rapamycin complex 1 (mTORC1) by cAMP response element binding protein (CREB)-mediated transcriptional regulation of the small GTPase Ras homologue enriched in brain (RHEB) (71). Additionally, Wu et al. suggested that PDK4 is essential for tumor necrosis factor alpha (TNF-α) to execute its pro-survival function *via* nuclear factor ‘kappa-light-chain-enhancer’ of activated B-cells (NF-*k*B), and consequently PDK4 deficiency in HCCa cells results in apoptosis (72).

In contrast, a tumor suppressive effect of PDK4 was observed in lung cancer (61, 73) and HCCa (16, 60). Sun et al. described that a metabolic switch from glycolysis to OXPHOS was observed in NSCLCa cells that underwent EMT, which was induced by knockdown of PDK4 (73). In the liver, PDK4 expression is associated with increased survival and liver function of patients undergoing liver resection due to colorectal liver metastases, and its downregulation predicted poor prognosis in HCCa patients (59). Besides that, loss of PDK4 resulted in enhanced lipogenesis and more aggressive tumors in HCCa (16). In PCa, Oberhuber et al. showed the association of low PDK4 with a risk of earlier disease recurrence in PCa, independent of tumor grading and tumor stage (63).

## PDK1–4 Are Associated With Therapy Resistance in Several Cancers

PDK1–4 have been associated with therapy resistance in several cancers. Qian et al. revealed that miR-4290 improved the sensitivity of GCa cells to cisplatin and induced apoptosis by downregulating PDK1 expression (38). Moreover, genetic knockdown of PDK1 abolished hypoxia-induced 5-fluorouracil (5-FU) resistance in GCa cells (74) and sensitized resistant OCa cells to cisplatin-induced cell death and apoptosis (75). Recently, PDK2 has been shown to induce resistance to 5-FU in chemo resistant CRCa cells (14), to be associated with cisplatin resistance in HNSCCa cells (49) and acquired paclitaxel-resistance in NSCLCa patients (66). PDK3 is associated with chemo resistance in GCa cells (68) and increased drug resistance in CoCa cells (15). Altered regulation of PDK4 is suggested to play a role in antiestrogen resistance in BrCa cells (57). In tamoxifen resistant MCF-7 breast cancer cells, *PDK4* mRNA overexpression, but not enhanced protein levels, have been shown (57). Wang et al. described PDK4-induced chemo resistance in OCa (62). Sun et al. showed that downregulation of PDK4 in lung cancer drives EMT and promotes erlotinib resistance in EGFR mutant lung cancer cells (73). The combination of chemotherapeutic drugs with dichloroacetate (DCA), a PDK inhibitor, have been shown to enhance therapeutic efficacy (76). In addition, DCA has been described to increase radiosensitivity by increasing tumor oxygenation and reactive oxygen species (ROS) activity (76).

The association of PDK to therapy resistance can be explained by the anti-apoptotic and ROS protective benefits of the Warburg effect, which results in proliferative advantages (27, 76). The Warburg effect is supported by activated oncogenes and HIF1α, which induce the expression of glycolytic enzymes and transporters, such as glucose transporters (GLUTs) or lactate dehydrogenase A (LDHA) that are involved in glucose uptake, lactate production, and lactate secretion (27, 77). As a result, tumors are characterized by high levels of glycolysis and lactate production, and low levels of PDC activity and OXPHOS (27, 77). High accumulation of lactate and low OXPHOS activity lead to reduced activation of the apoptotic cascade and ROS, which protect cancer cells from cytotoxic effects of oxidative damage and apoptosis (3, 9, 10, 27, 76). Although aerobic glycolysis generates less energy (2 ATP per glucose molecule) than OXPHOS (36 ATP per oxidized glucose molecule) a high rate of glucose uptake of the tumor can compensate the tumor’s energetic demands (10, 11). DeBerardinis et al. suppose that generation of energy *via* glycolysis is faster and therefore more attractive than the more energy efficient but slower OXPHOS (77).

## The Peculiar Energy Metabolism of PCa and its Implications on the Role of PDK4

**Figure 2:**
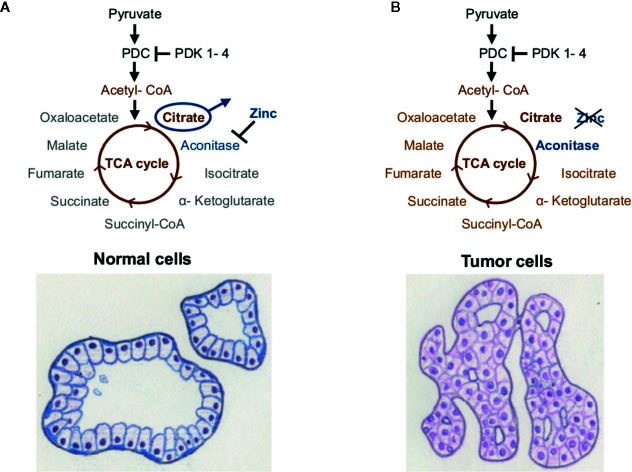
Energy metabolism of the prostate. **(A)** Healthy prostate cells accumulate high amounts of zinc, which inhibit the enzyme m-aconitase and thereby truncate the TCA cycle. **(B)** Prostate tumor cells show lower levels of zinc, whereby the enzyme aconitase remains active and citrate can be metabolized *via* the TCA cycle and OXPHOS. Taken from (5), inspired by (78).

Primary PCa is lacking the Warburg effect and has a very distinctive energy metabolism compared to most other cancer types, showing high TCA cycle/OXPHOS activity (78, 79). The energy metabolism of the normal prostate cell is a result of its biological function, where the glandular epithelial cells secrete prostatic fluid and its main component—citrate—into the lumen (79, 80). Prostate epithelial cells accumulate extensive amounts of zinc, which inhibit the TCA cycle enzyme m-aconitase (78–80). Thereby citrate cannot be converted to isocitrate, the TCA cycle is truncated, and citrate is secreted by the prostatic epithelial cells (**Figure 2A**). As the prostatic epithelial cells have low OXPHOS activity, they mainly rely on aerobic glycolysis and are therefore energetically inefficient (78–80). In contrast, PCa cells no longer present zinc-accumulation and citrate-secretion, but activated TCA cycle/OXPHOS, thereby generating additional ATP (78, 80) (**Figure 2B**). Latonen et al. and Xue et al. show an increase in aconitase expression in PCa cells compared to non-cancerous tissue, which indicates their citrate oxidizing ability (81, 82). Acetyl-CoA provided by the TCA cycle serves as substrate for lipogenesis, which is known to be hyper-activated in PCa and associated with androgen resistance and tumor aggressiveness (17, 83–85). Lipogenic enzymes, as well as genes involved in cholesterol synthesis, have been shown to be regulated by androgen signaling (84). In turn, inhibition of fatty acid synthase (FASN), a key enzyme of *de novo* fatty acid synthesis, led to reduced androgen receptor (AR) expression in castration-resistant PCa (CRPCa) (85). PDK4 is not only a regulator of PDC activity, but can also alter fatty acid metabolism, as has been shown in HCCa cells (16). Here, knockdown of PDK4 did not alter OXPHOS, but resulted in enhanced expression of FASN and stearoyl-CoA desaturase (SCD) (16). Similarly, PDK4 was shown to enhance lipogenesis in lung cancer cells (61).

Since low levels of PDK4 result in enhanced OXPHOS and/or enhanced lipogenesis, both of which are associated with poor prognosis in PCa, PDK4 should have a tumor suppressive effect in primary PCa. Recently, a protective effect of high *PDK4-* expression in PCa in a transcriptomic patient dataset was demonstrated (63). In accordance with these data, augmented gene expression and protein levels of the PDC subunit E1 (*PDHA1*) and the PDC activator PDP1 were identified in PCa (86). In contrast, Wang et al. reported lower proliferation and increased apoptosis in PCa cells upon knockdown of all PDK isoforms (40). While PDK4 and PDK2 are expressed at lower levels, PDK1 and PDK3 are supposedly overexpressed in PCa and associated with advanced tumor stages (40).

The specific effects of PDK1–4 on PCa energy and fatty acid metabolism have not been investigated yet. In addition to their direct implications on pyruvate provided carbon use and OXPHOS, also compensative mechanisms must be considered. These were shown to be active in metformin treated PCa cells (87). Here, metformin reduces entry of glucose-derived carbon into the TCA cycle due to complex I inhibition (87). The loss of glucose as carbon source was compensated by increased reductive glutamine metabolism, which provides α-ketoglutarate to the TCA cycle (87). Additional inhibition of the reductive glutamine pathway resulted in enhanced PCa cell sensitivity to metformin (87).

Given the importance of the hyperactive FASN and the unique dependence on OXPHOS in primary PCa, we are convinced that the action of PDK4 in PCa has a profound clinical significance and therefore requires immediate research.

## Summary and Conclusion

PDK1–3 are described as oncogenes in different cancer types where their high expression is associated with EMT and metastasis, higher proliferation and migration, and most relevantly with therapy resistances, such as to 5-FU in CRCa and GCa, cisplatin in HNSCCa and OCa or paclitaxel in NSCLCa. In contrast to PDK1–3, data suggest either oncogenic or tumor suppressive function of PDK4, dependent on the metabolic profile of the tumor. It acts as an oncogene and is linked to therapy resistance in tumors that benefit from high glycolytic activity, such as in BrCa, AML, CoCa, OCa and BlCa. However, PDK4 can act as tumor suppressor in cancers that depend on high OXPHOS activity and/or high amounts of TCA cycle intermediates, as has been shown in PCa. Also in NSCLCa and HCCa low PDK4 levels are described to lead to more aggressive tumors and therapy resistance. We conclude that the combinatorial treatment of DCA with chemotherapeutic drugs might enable overcoming therapy resistances only in cancer types with a fitting metabolic profile (76). A large body of research is directed to tumors that profit from high glycolysis/lactate accumulation, whereas far less is known about those cases, where high OXPHOS contributes to tumor aggressiveness. Especially for PCa, where only little research is available on the mechanistic regulation and effects of PDK4, more research is needed in this regard.

## Author Contributions

All authors contributed to the conception and design of the review. EA and MO conducted literature research. EA wrote the first draft of the manuscript. All authors contributed to editing and rewriting of the manuscript. EA and MO created the figures for the manuscript. All authors contributed to the article and approved the submitted version.

## Funding

EA and LK are funded by the Austrian Science Fund (FWF): IPPTO project number DOC 59-B33. MO and LK were funded by the COMET Competence Center CBmed-Center for Biomarker Research in Medicine (FA791A0906.FFG). The COMET Competence Center CBmed is funded by the Austrian Federal Ministry for Transport, Innovation and Technology (BMVIT); the Austrian Federal Ministry for Digital and Economic Affairs (BMDW); Land Steiermark (Department 12, Business and Innovation); the Styrian Business Promotion Agency (SFG); and the Vienna Business Agency. The COMET program is executed by the FFG. LK was in addition funded by the FWF grant P26011 and the Christian-Doppler Lab for Applied Metabolomics. The financial support by the Austrian Federal Ministry for Transport, Innovation and Technology and the National Foundation for Research, Technology and Development is gratefully acknowledged.

## Conflict of Interest

LK is a member of the scientific advisory board of CBmed-Center for Biomarker Research in Medicine GmbH. Author MO was employed by COMET centre (K1) CBmed—Center for Biomarker Research in Medicine GmbH.

The remaining authors declare that the research was conducted in the absence of any commercial or financial relationships that could be construed as a potential conflict of interest.

## Abbreviations

5-FU, 5-fluorouracil; ACAT1, Acetyl-CoA acetyltransferase 1; AML, Acute myeloid leukemia; AR, Androgen receptor; ATP, Adenosine triphosphate; BrCa, Breast cancer; BlCa, Bladder cancer; ChCa, Cholangiocarcioma; CoA, Coenzyme-A; CoCa, Colon cancer; CRCa, Colorectal cancer; CRPCa, Castration resistant prostate cancer; CREB, CAMP response element binding protein; DCA, Dichloroacetate; E1, Pyruvate dehydrogenase; E2, Dihydrolipoamide acetyl-transferase; E3, Dihydrolipoamide dehydrogenase; E3BP, E3 binding protein; EMT, Epithelial–mesenchymal transition; FASN, Fatty acid synthase; FGFR1, Fibroblast growth factor receptor 1; GCa, Gastric cancer; GLUT, Glucose transporters; HCCa, Hepatocellular carcinoma; HIF1*α*, Hypoxia inducible factor 1 alpha; HNSCCa, Head and neck squamous cell carcinoma; LDHA, Lactate dehydrogenase A; mTORC1, Mechanistic target of rapamycin complex 1; NAD+, Nicotinamide adenine dinucleotide; NADH, Reduced form of NAD+; NF-*k*B, Nuclear factor kappa-light-chain-enhancer; NSCLCa, Non-small cell lung cancer; OCa, Ovarian cancer; OXPHOS, Oxidative phosphorylation; PCa, Prostate cancer; PDK, Pyruvate dehydrogenase kinase; PDP, Pyruvate dehydrogenase phosphatase; RHEB, Ras homolog enriched in brain; ROS, Reactive oxygen species; SIRT, Sirtuin; STAT3, Signal transducer and activator of transcription 3; SCD, Stearoyl-CoA desaturase; TCA cycle, Tricarboxylic acid cycle; TNF, Tumor necrosis factor.
